# The Nimble Stage 1 Study Validates Diagnostic Circulating Biomarkers for Nonalcoholic Steatohepatitis

**DOI:** 10.21203/rs.3.rs-2492725/v1

**Published:** 2023-01-19

**Authors:** Arun Sanyal, Sudha Shankar, Katherine Yates, James Bolognese, Erica Daly, Clayton Dehn, Brent Neuschwander-Tetri, Kris Kowdley, Raj Vuppalanchi, Cynthia A. Behling, James Tonascia, Anthony Samir, Claude Sirlin, Sarah Sherlock, Kathryn Fowler, Helen Heymann, Tania Kamphaus, Rohit Loomba, Roberto Calle

**Affiliations:** Virginia Commonwealth University School of Medicine; Astrazeneca; Bloomberg School of Public Health, Johns Hopkins University; Cytel, Inc.; Cytel, Inc.; P-Value; Saint Louis University; Liver Institute Northwest; Indiana University School of Medicine; Sharp Memorial Hostipal; Bloomberg School of Public Health, Johns Hopkins University; Harvard School of Public Health; University of California at San Diego; Pfizer; University of California San Diego School of Medicine; Foundation for the NIH; Foundation for the NIH; University of California at San Diego; Regeneron Pharmaceuticals

**Keywords:** Nonalcoholic fatty liver disease (NAFLD), Nonalcoholic steatohepatitis (NASH), NAFLD activity score (NAS), Fibrosis stage, cirrhosis, steatohepatitis, fibrosis, biomarkers, diagnostic panel, context of use, validation

## Abstract

**Background:**

There are no approved noninvasive tests (NIT) for the diagnosis of nonalcoholic steatohepatitis (NASH) and its histological phenotypes.

**Methods:**

The FNIH-NIMBLE consortium tested 5 serum-based NIT panels for the following intended uses: NIS4: At-risk NASH, a composite of NASH with NAFLD activity score (NAS) ≥ 4 and fibrosis stage ≥ 2, OWLiver: NASH and NAS ≥ 4, enhanced liver fibrosis (ELF), PROC3 and Fibrometer VCTE: fibrosis stages ≥ 2, ≥ 3 or 4. Aliquots from a single blood sample obtained within 90 days of histological confirmation of NAFLD were tested. The prespecified performance metric tested for was a diagnostic AUROC greater than 0.7 and superiority to ALT for diagnosis of NASH or NAS ≥ 4 and to FIB-4 for fibrosis.

**Results:**

A total of 1073 adults including NASH (n = 848), at-risk NASH (n = 539) and fibrosis stages 0–4 (n = 222, 114, 262, 277 and 198 respectively) were studied. The AUROC of NIS4 for at-risk NASH was 0.81 and superior to ALT and FIB4 (p < 0.001 for both). OWliver diagnosed NASH with sensitivity and specificity of 77.3% and 66.8% respectively. The AUROCs (95% CI) of ELF, PROC3 and Fibrometer VCTE respectively for fibrosis were as follows: ≥ stage 2 fibrosis [0.82 (0.8–0.85), 0.8 (0.77–0.83), and 0.84 (0.79–0.88)], ≥ stage 3 [0.83 (0.8–0.86), 0.76 (0.73–0.79), 0.85 (0.81–0.9), stage 4 [0.85 (0.81–0.89), 0.81 (0.77–0.85), 0.89 (0.84–0.95)]. ELF and Fibrometer VCTE were significantly superior to FIB-4 for all fibrosis endpoints (p < 0.01 for all).

**Conclusions:**

These data support the further development of NIS4, ELF and Fibrometer VCTE for their intended uses.

## Introduction

Nonalcoholic fatty liver disease (NAFLD) is a leading cause of liver-related morbidity and mortality ^1^. The presence of nonalcoholic steatohepatitis (NASH) an active form of NAFLD and fibrosis stage of 2 or higher is linked to increased risk of liver outcomes and death ^2–4^. Identification of such individuals and targeting them for therapeutic intervention is a cornerstone of clinical assessment and inclusion in clinical trials ^5^.

Histological evaluation of liver biopsy sections is the reference standard for assessment of NASH but requires an invasive liver biopsy with its associated risks which has limited its widespread use ^6–8^. This has spurred much work to establish non-invasive tests (NITs) to diagnose NASH and fibrosis, yet none have met the evidence burden needed for regulatory approval. Validation of such NITs to regulatory standards remains a major unmet need for the field.

The **N**on-**I**nvasive **B**ioMarkers of **M**eta**B**olic **L**iver Diseas**E** (NIMBLE) consortium was established by the Foundation-NIH (FNIH) to generate evidence to support advanced regulatory qualification of one or more NITs for the evaluation of nonalcoholic fatty liver disease (NAFLD) ^9^. The current study represents a collaborative effort between the NIMBLE consortium and the National Institute of Diabetes and Digestive and Kidney Diseases-NASH Clinical Research Network (CRN). The objective was to perform a cross-sectional study to rigorously define the sensitivity and specificity of five serum-based NIT panels for the diagnosis of one or more of the following: NASH, high histological NAFLD activity and subpopulations with clinically relevant stages of fibrosis including cirrhosis. The panels were pre-selected on the basis of their analytic robustness, available literature and potential for scale-up for widespread use. The final results of this study are presented below.

## Materials And Methods

Serum samples collected from adult participants with NAFLD in a non-interventional registry (database 1 and 2 (DB1 and DB2) and baseline samples from clinical trials (PIVENS and FLINT) across 12 NIDDK NASH CRN clinical sites (*supplemental Table 1*) were analyzed. Participants were enrolled across these studies from 2004-2017 and provided informed consent at enrollment; the use of de-identified samples and meta-data was considered exempt from additional consenting requirements. The investigators have analyzed the data and take responsibility for the contents of this manuscript. The studies were done in accordance to STARD guidance and reported using the TRIPOD statement ^10,11^.

### Context of Use

In individuals with NAFLD or with risk-factors for NAFLD, to serve as a diagnostic enrichment tool for the identification of various histological phenotypes of NAFLD, intended for selection for participation in NAFLD/NASH clinical trials and/or drug treatment. Those who were overweight or obese, or had other features of metabolic syndrome were considered to be at risk for NAFLD ^12^. The presence of specific phenotypes to be diagnosed included:

- At risk NASH: (NASH + NAFLD activity score (NAS) ≥ 4 + fibrosis stage 2 or higher)^9^
- Nonalcoholic steatohepatitis (borderline or definite)
- A NAS ≥ 4
- Clinically significant fibrosis (fibrosis stage ≥ 2)
- Advanced fibrosis (stage 3 or 4)
- Cirrhosis (stage 4)

### Study Design

#### A: Study population

The study population was curated from the CRN patient base to ensure sufficient number of individuals with and without the histological phenotypes of interest and a balanced distribution of fibrosis stages to avoid spectrum bias. The current analysis included aliquots from a serum-sample obtained within 90-180 days of an evaluable liver biopsy demonstrating NAFLD. For Fibrometer VCTE (vibration-controlled transient elastography), a liver stiffness measurement was required within 180 days of the biopsy. Exclusion criteria included pregnancy at the time of sample collection or biopsy, co-morbid liver diseases, use of drugs known to cause steatosis, non-availability of minimum required serum, bariatric surgery within 3 years prior to biopsy, prior liver transplant and known primary or secondary malignancy of the liver.

#### B: Biomarker panels tested and their intended context of use

Serum biomarker panels selected by the NIMBLE circulating workstream were reviewed and approved by the project team, NASH CRN ancillary study and steering committees. These included:

NIS4^13^: mir34a, Hemoglobin A1c, α2-macroglobulin, YKL-40

OWliver^14^: triglyceride species with variable number of saturated fatty acids

ELF test^15^: type III procollagen peptide, hyaluronic acid and TIMP-1

PROC3^16^: procollagen-3 fragment reflective of fibrogenesis

Fibrometer VCTE ^17^: LSM, age, gender, α2-macroglobulin, INR, platelet count, AST, GGT

The intended use of NIS4 was to diagnose at-risk NASH and its components whereas the OWliver panels intended use was to diagnose the presence of NASH (*supplemental Table 2*). The intended uses of the ELF test, PROC3 and Fibrometer VCTE were to diagnose clinically significant fibrosis (≥ stage 2 fibrosis), advanced fibrosis (≥ stage 3 fibrosis) or cirrhosis (stage 4 fibrosis).

#### C: Study Approach

The study plan was summarized in a letter of intent approved by the US federal government Food and Drug Administration ^9,18^. De-identified, bar-coded frozen aliquots of the same serum sample from each participant without any prior freeze-thaw were released to the individual laboratories. These laboratories generated panel scores which were provided to the independent statistical team (Cytel) who deposited these in the CRN data warehouse. The CRN then released the meta-data linked to the bar codes to Cytel who implemented the prespecified statistical analysis plan without involvement of individual vendors whose panels were tested. The NIMBLE circulating workstream and statistical team then jointly reviewed the results and interpreted the data.

#### D: Histological Examination:

The pathology committee of the NASH CRN performed the histological assessment, masked to clinical and laboratory data, using an established and validated protocol ^19,20^. The key measures included the presence of steatohepatitis and individual severity grades for steatosis (0-3), lobular inflammation (0-2), hepatocellular ballooning (0-2) and fibrosis stage (0-4). The NAS was computed from the scores for steatosis, ballooning and inflammation while “at risk” NASH was computed from the presence of its components ^9,20^.

### Statistical Plan

There were two pre-specified performance metrics which formed the basis for hypothesis-testing. First, that the area under receiver operating curve (AUROC) for each panel would be 0.7 or higher for its intended use with 95% confidence limits that would not intersect 0.5. Next, the biomarker performance would be superior to commonly used blood-based laboratory aids for their intended use. The AUROC of each panel was therefore compared to that of ALT for diagnosis of NASH or NAS≥ 4 and FIB-4, a commonly used laboratory aid based on age, AST, ALT and platelet counts, for diagnosis of fibrosis severity ^21,22^. The sensitivity and specificity were computed at the Youden cut-point. The sensitivity was further estimated keeping specificity fixed at 90% and conversely specificity was measured keeping the sensitivity fixed at 90%. Finally, the positive and negative predictive values were computed at various prevalence of specific NAFLD phenotypes. Missing data were assumed missing at random from the statistical analysis, as they resulted from sample handling and laboratory issues independent of the relationship between biomarkers and histology; complete case-analysis was done.

The sample size was estimated to detect a difference of at least 0.05 between the AUROC of FIB-4 or ALT and the relevant biomarker panel with a power of at least 80% with a one-sided p value of 0.025. It was assumed that the AUROC for FIB-4 would be 0.8 for fibrosis. Additionally, due to potential correlation between FIB-4 or ALT versus the biomarker panels, adjustments were made assuming the correlation coefficients ranging from 0.5-0.8. Based on these a total number of participants needed with NASH and fibrosis stage 2 or 3 versus 0 or 1 was 400 each. For analysis of cirrhosis, 180 individuals with cirrhosis were needed.

## Results

**Figure 1:**
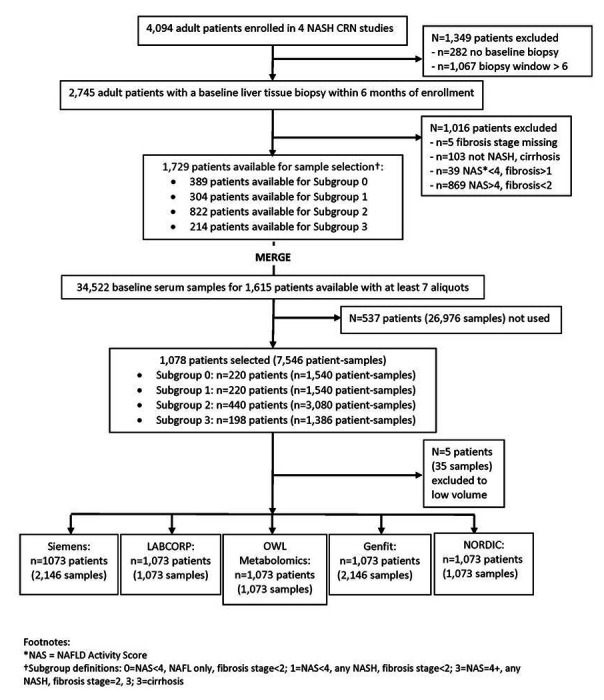
Sample derivation from the NASH CRN cohort and their use for various tests *NAS = NAFLD Activity Score †Subgroup definitions: 0=NAS<4, NAFL only, fibrosis stage<2; 1 =NAS<4, any NASH, fibrosis stage<2; 3=NAS=4+, any NASH, fibrosis stage=2, 3; 3=cirrhosis

The NASH CRN cohort had 4094 participants (Figure 1). A total of 2479 individuals were excluded because of age, lack of samples or evaluable liver biopsies. Of the remaining individuals, consecutive patients for each stage of disease were selected to ensure that that enough patients were available to meet sample size estimates and to have a relatively balanced-distributed spectrum of fibrosis severity (stages 0-4, n= 222, 114, 262, 277 and 198 respectively). A total of 1073 individuals meeting eligibility criteria were thus included for this analysis with 90% of individuals having a serum sample within 90 days of the liver biopsy (Table 1).

The mean age of the cohort was 54 years and was preponderantly female and white. NAFL was present in 225 individuals while 835 had NASH and 13 an indeterminate NAFLD phenotype. Those without fibrosis were younger, had mainly had NAFL and lower NAS compared to those with fibrosis stage 2 or higher. The study population for Fibrometer VCTE was a smaller subset of the larger population (n= 396) for this analysis due to lack of availability of a VCTE examination within 6 months of the liver biopsy in many individuals. The baseline features of this subset were similar to the larger cohort (*supplemental Table 3*).

### At-risk NASH

NIS4 was the only panel with an intended use to diagnose underlying composite phenotype of “at risk” NASH (n=539) as defined above. The sensitivity and specificity were 78.1% and 73.6% respectively with an AUROC of 0.815 at the optimal cut-point (Table 2). The AUROC was superior (p< 0.001 for both) to both ALT (AUROC 0.726) and FIB-4 (AUROC 0.704).

### NASH Diagnosis

**Figure 2:**
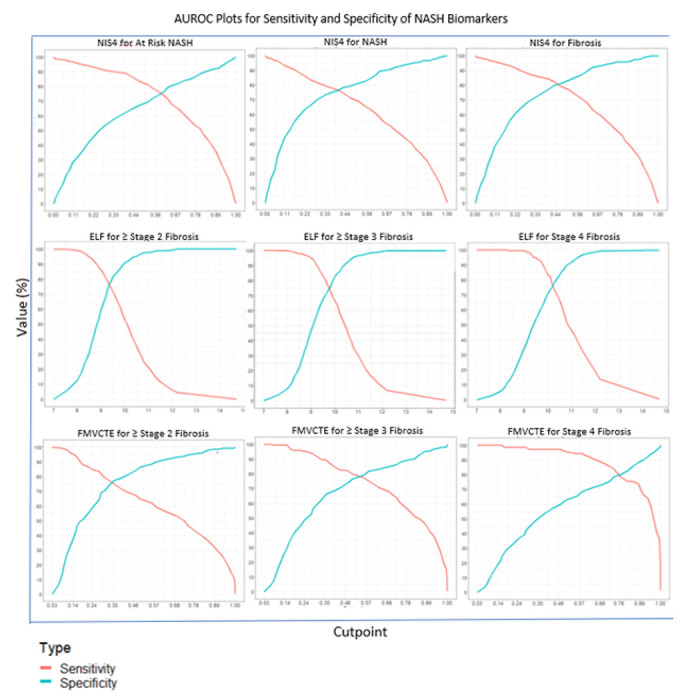
Sensitivity and Specificity of key NIT panels for their respective intended uses are shown as a function of the cutoff scores for the NIT. The top panel demonstrate changes in sensitivity and specificity at varying NIS4 cutoff scores for the diagnosis of at-risk NASH (panel A) and its key subcomponent diagnosis of NASH (panel B) and stage 2 or greater fibrosis (Panel C). The middle panels show similar data for the ELF test for the diagnosis of ≥ stage 2 fibrosis (panel D), ≥stage 3 (panel E) and stage 4 i.e. cirrhosis (panel F). The lower panel demonstrates the changes in sensitivity and specificity at varying Fibrometer VCTE (FM-VCTE) score cutoffs for the diagnosis of ≥ stage 2 fibrosis (panel G), ≥ stage 3 fibrosis (panel H) and stage 4 fibrosis (panel I). Individual plots were derived from 50 individual score cutoffs covering the range where sensitivity was 100% to where specificity approached 100% followed by smoothening of the graph to cover the dynamic range of scores for their intended uses.

NIS4 and the OWLiver tests had an intended use to diagnose NASH (*supplemental Table 2*). NIS4 (Youden cut-point 0.539) had an AUROC of 0.83 (95% CI 0.8-0.86) and was superior to ALT (AUROC 0.67) for this intended use (Table 2, Figure 2). The sensitivity and specificity were 77.7% and 76.2% respectively at this cut-point. NIS4 had a specificity of 47.7% and sensitivity of 54.4% when sensitivity and specificity were constrained at 90% respectively (*supplemental table 4*) and both were significantly superior to ALT (p<0.001 for both). The OWLiver provided the results in categorical format which did not permit generation of an AUROC; it diagnosed NASH with a sensitivity of 77.3%. and specificity of 66.8%.

### High NAFLD Activity Score (NAS ≥ 4)

The AUROC (0.815, 95% CI 0.786-0.844) for NIS4 was significantly superior to ALT (AUROC 0.726, sensitivity 71.1%, specificity 64.1%) the comparator for panels intended to diagnose high activity (p<0.001). The specificity and sensitivity of NIS4 were 57.8% and 46.2% when sensitivity and specificity were locked at 90% respectively and both were significantly superior to ALT (p< 0.001 for both).

### Clinically significant fibrosis (Fibrosis stage ≥ 2):

NIS4, ELF, PROC3 and Fibrometer VCTE had an intended use to identify clinically significant fibrosis in those with NAFLD. The AUROCs of NIS4, ELF, PROC3 and Fibrometer VCTE were 0.874, 0.828, 0.8 and 0.841 respectively. Their respective sensitivity and specificity at their Youden index are provided in Table 2. FIB-4 had an AUROC of 0.798 very close to the expected AUROC of 0.8 ^22^. NIS4 (p< 0.001), ELF (p< 0.01) and Fibrometer VCTE (p< 0.001) were all superior to FIB4. Similar data were obtained when the performance of these panels with sensitivity and specificity constrained at 90% were evaluated (*supplemental Table 4*).

### Advanced fibrosis (Stages ≥ 3):

The operational definition of advanced fibrosis included those with stage 3 or 4. The AUROCs of the panels tested for the diagnosis of advanced fibrosis were as follows: FIB-4 (0.789), ELF (0.835, p<0.001 vs FIB4), PROC3 (0.809, p=n.s. vs FIB-4), Fibrometer VCTE (0.841, p< 0.001 vs FIB4). A secondary analysis of NIS-4 for advanced fibrosis provided an AUROC of 0.78, p=n.s. vs FIB4). The sensitivity (ELF 50.3% and Fibrometer VCTE 54.2%) and specificity (ELF 55.3% and Fibrometer VCTE 59.6%) with specificity and sensitivity fixed at 90% respectively were both statistically superior to FIB-4 (*supplemental Table 4*).

### Cirrhosis (stage 4)

The AUROCs for the diagnosis of cirrhosis were 0.81 for FIB-4, 0.855 for ELF (p< 0.001 vs FIB-4) and 0.897 for Fibrometer VCTE (p=0.002 vs FIB-4). The sensitivity of ELF and Fibrometer VCTE at the Youden index were 82.1% and 94.2% while the specificities were 73.3% and 70.4% respectively. Their performance at 90% sensitivity (specificity: ELF 60.5%, Fibrometer VCTE 72.5%) and 90% specificity (Sensitivity: ELF 49%, Fibrometer VCTE: 66.7%) were also statistically superior to FIB-4 (*Supplemental Table 4*).

## Discussion

The regulatory path for approval of a diagnostic test requires rigorously established sensitivity and specificity in a study cohort that is both powered and balanced with respect to the presence or absence of the condition being studied. The current study establishes this first step and is the foundation for the use of specific cut-points in relevant populations in the next stage towards regulatory approval of these diagnostic enrichment tools for NASH^18^.

The study has several methodological strengths. The time from biopsy to blood draw was short and all analyses including the comparators were made on the same blood sample. Further, all samples were drawn, aliquoted, stored and analyzed without multiple freeze-thaw using prespecified protocols. Histology was read independently using a rigorous pre-specified protocol by the pathology committee of the NASH CRN masked to clinical and laboratory data. The distribution of fibrosis stages in the cohort avoided spectrum bias. Finally, for each of the phenotypes studied, the sample size included enough number of individuals with and without the phenotype assuring power both for sensitivity and specificity.

The practical application of these data has to be considered in the context of how the tests are used. In primary care where the prevalence of advanced fibrosis is 1%, positive tests are likely to be false positives and even with excellent sensitivity and specificity the PPV will be low ^23^. Using these tests to identify patients for clinical trials in such settings are likely to have many false positives resulting in high screen fail rates. The NPV for FIB4 as well as all of the biomarker panels (ranged from 98-99.7% when the population prevalence of advanced fibrosis was 1% (*supplemental Table 5*). These tests can therefore be applied for exclusion of this phenotype for both clinical management and for screening for trials of at-risk NASH in a primary care setting.

The prevalence of at-risk NASH or its subsets NASH with advanced fibrosis or cirrhosis in hepatology clinic settings are higher and range from 10–40% ^2,12,24^. It is encouraging to note that the high NPV in settings with low prevalence was maintained at these ranges while the positive predictive values approached 80% at the 40% prevalence when the Youden cut point was used (*supplemental Table 5*). In clinical trial settings, these data should allow exclusion of those without these phenotypes while limiting overdiagnosis compared to a primary care setting. Additional enhancement of certainty for ruling in disease by using the cut point for 90% specificity will however be associated with a loss of sensitivity and attendant misclassification.

Further improvement is likely to require an algorithmic approach using multiple panels or use of imaging-based tests for greater precision in identification of this population. Recently, MR-elastography with FIB4 or AST has been shown to identify those with NASH and advanced fibrosis or at-risk NASH respectively and may provide such tools ^25–27^. The current data can’t however be directly compared to these due to methodological differences.

For those with advanced fibrosis or cirrhosis, a mistaken diagnosis of absence of these phenotypes may cause patients to be followed without surveillance for hepatocellular cancer or varices. The overall high NPVs suggest that the risks are in general low. Conversely, overdiagnosis due to the modest PPVs may result in futile additional testing including liver biopsies with its attendant risks. ELF and Fibrometer VCTE can identify 82–94% of true positive cases of cirrhosis but also may over-diagnose some patients to have cirrhosis in clinics with high prevalence of cirrhosis (Table 5). The risks of overdiagnosis has to be considered in the context of the risks of missing advanced fibrosis or cirrhosis altogether in specific populations both in clinical practice and for consideration for inclusion in trials.

This study also has some limitations. The NASH CRN is based at tertiary care centers generating ascertainment bias. The study population is predominantly white and the data are not generalizable to other races. Despite these limitations, the current study provides a rigorous evidence base to establish the sensitivity and specificity of these biomarker panels serves as a critical step in the ultimate regulatory approval of some of these panels as diagnostic enrichment tools in clinical practice and to determine eligibility for inclusion in clinical trials for NASH.

## Figures and Tables

**Table 1:** Demographic, Clinical and Laboratory Data from the Study Cohort

	Stage 0	Stage 1	Stage 2	Stage 3	Stage 4
	N = 222	N = 114	N = 262	N = 277	N = 198

Age (years)	47.8 (12.2)	48.1 (13.8)	51.7 (11.5)	54.4 (11.2)	56.2 (9.8)
Gender (males) [n (%)]	99 (44.6%)	52 (45.6%)	102 (38.9%)	91 (32.9%)	60 (30.3%)

White [n (%)]	158 (71.2%)	68 (59.6%)	199 (76.2%)	217 (78.9%)	169 (86.2%)
African American [n (%)]	4 (1.8%)	5 (4.4%)	8 (3.1%)	8 (2.9%)	3 (1.5%)
Hispanic [n (%)]	34 (15.3%)	20 (17.5%)	28 (10.7%)	24 (8.7%)	16 (8.2%)
Other [n (%)]	26 (11.7%)	21 (18.4%)	26 (10.0%)	26 (9.5%)	8 (4.1%)

BMI (kg/m^2^)	32.8 (6.6)	33.3 (6.1)	34.5 (6.3)	36.1 (6.6)	36.4 (7.3)
Waist circ (cm)	104.7 (14.7)	107.3 (13.9)	110.8 (14.2)	114.3 (14.8)	113.7 (15.1)
Type 2 diabetes [n (%)]	45 (20.3%)	41 (36.0%)	113 (43.1%)	162 (58.5%)	129 (65.2%)
Hypertension [n (%)]	94 (42.3%)	65 (57.0%)	164 (62.6%)	191 (69.0%)	132 (66.7%)

AST (IU/L)	27.8 (13.3)	31.9 (17.7)	50.3 (29.3)	58.3 (39.8)	51.9 (28.9)
ALT (IU/L)	38.5 (25.4)	45.0 (34.6)	65.5 (43.1)	68.1 (47.8)	49.1 (34.5)
ALP (IU/L)	86.6 (30.5)	80.6 (28.2)	87.0 (28.0)	93.0 (33.2)	114.5 (53.2)
Total Bilirubin (mg/dL)	0.5 (0.3)	0.6 (0.5)	0.5 (0.3)	0.5 (0.4)	0.8 (0.8)
INR	1.0 (0.1)	1.0 (0.2)	1.0 (0.1)	1.1 (0.1)	2.8 (4.3)
Albumin (g/dL)	4.6 (0.3)	4.6 (0.3)	4.6 (0.3)	4.5 (0.3)	4.3 (0.4)

Hemoglobin (g/dL)	14.4 (1.4)	14.3 (1.5)	14.2 (1.5)	13.9 (1.5)	13.6 (1.6)
WBC (10^3^/μL)	7.0 (2.2)	7.1 (2.0)	7.3 (2.0)	7.6 (6.0)	6.5 (3.3)
Platelet (cells/mL)	250.3 (62.7)	243.5 (82.9)	237.8 (60.5)	218.7 (66.2)	165.9 (64.1)

Fasting glucose (mg/dL)	101.3 (33.8)	106.7 (42.0)	114.6 (42.2)	116.6 (34.8)	126.2 (51.7)
Fasting insulin (μU/mL)	17.8 (14.2)	25.5 (32.4)	26.8 (29.1)	30.9 (27.6)	35.5 (35.4)
Total cholesterol (mg/dL)	193.7 (43.1)	181.2 (43.3)	189.7 (48.5)	183.3 (42.3)	174.2 (40.4)
LDL-C (mg/dL)	117.5 (36.5)	105.9 (36.6)	112.0 (39.2)	106.1 (38.1)	100.7 (35.3)
HDL-C (mg/dL)	45.1 (10.9)	44.4 (13.5)	42.9 (11.9)	42.8 (11.9)	45.2 (13.2)
Triglycerides (mg/dL)	169.8 (108.0)	168.1 (108.5)	203.1 (275.0)	186.3 (114.9)	141.5 (66.2)

Statin Use [n (%)]	63 (28.4%)	46 (40.4%)	91 (34.7%)	113 (40.8%)	84 (42.4%)

Time from biopsy to study entry (days)	55.16(24.32)	60.44(26.93)	53.60(25.24)	53.18(24.15)	79.31(39.22)

NAFL [n (%)]	195 (87.8%)	23 (20.2%)	0 (0.0%)	0 (0.0%)	7 (3.5%)
NASH [n (%)]	27 (12.2%)	91 (79.8%)	262 (100%)	277 (100%)	178 (89.9%)
Steatosis grade	1.3 (0.5)	1.2 (0.4)	2.0 (0.8)	1.8 (0.9)	1.3 (0.9)
Ballooning grade	0.1 (0.3)	0.3 (0.5)	1.2 (0.7)	1.5 (0.7)	1.5 (0.7)
Lobular Inflammation	1.0 (0.3)	1.1 (0.3)	1.7 (0.7)	1.8 (0.7)	1.5 (0.7)
Portal inflammation	0.8 (0.6)	0.9 (0.5)	1.2 (0.5)	1.4 (0.5)	1.7 (0.5)
NAS	2.5 (0.6)	2.5 (0.6)	4.8 (1.5)	5.2 (1.6)	4.2 (1.6)

All statistics presented are means (standard deviations), unless otherwise specified.

* Time between the liver biopsy and study enrollment for 109 (10%) of the cohort was between 92-183 days.

**Table 2:** Sensitivity and Specificity of individual panels for their intended use

	Sensitivity(%)	Specificity(%)	Youden index	AUROC (95% CI)	Significance (vs ALT or FIB4)

**NASH Diagnosis**					
ALT	63.2	64.8	0.28	0.678[0.639,0.717]	
NIS4	77.7	76.2	0.539	0.832[0.801,0.864]	<0.001
OWL	77.3	66.8	Categorical Data AUROC can’t be computed		

**NAS ≥ 4**					
ALT	71.1	64.1	0.352	0.726[0.694,0.759]	
NIS4	78.1	73.6	0.517	0.815[0.786,0.844]	<0.001

**At risk NASH**					
ALT	71.1	64.1	0.352	0.726[0.694.0.759]	
FIB4	76.4	58.4	0.349	0.704[0.671,0.737]	
NIS4	78.1	73.6	0.517	0.815[0.786.0.844]	<0.001

**Fibrosis stage ≥ 2**					
FIB4	65.6	80.6	0.462	0.798[0.768,0.828]	
ELF test	71.8	81.5	0.533	0.828[0.08,0.857]	0.013
NIS4	82.3	79.9	0.622	0.874[0.848,0.899]	<0.001
PROC3	69.8	81	0.507	0.809[0.779,0.839]	0.279
Fibrometer VCTE	66.7	86.4	0.53	0.841[0.796,0.886]	<0.001

**Fibrosis stage ≥ 3**					
FIB4	70.3	72.4	0.427	0.789[0.758,0.819]	
ELF	80.8	70.2	0.509	0.835[0.807,0.863]	<0.001
NIS4	72.9	74.8	0.476	0.788[0.757,0.820]	0.615
PROC3	71.4	71.4	0.428	0.764[0.732,0.795]	0.947
Fibrometer VCTE	76.2	81.3	0.575	0.858[0.814,0.902]	<0.001

**Fibrosis stage 4**					
FIB4	84.7	62.9	0.476	0.810[0.770,0.850]	
ELF	82.1	73.3	0.555	0.855[0.818,0.892]	<0.001
NIS4	78.1	61.4	0.395	0.725[0.681,0.760]	1
PROC3	66.2	68.5	0.346	0.728[0.685,0.770]	1
Fibrometer VCTE	94.2	70.4	0.646	0.897[0.843,0.951]	0.002

* for NASH diagnosis, NAS ≥ 4, significance is computed from comparison to AUROC for ALT

** for “at risk” NASH, the significance values are against AUROCS for both ALT and for FIB4

*** for fibrosis cutoffs, significance is computed from comparison to AUROC for FIB4

## Data Availability

Included in supplemental materials
